# Compliance of Primary Healthcare Workers in Saudi Arabia With the National Surveillance System of Tropical and Non-tropical Dermatological Diseases

**DOI:** 10.7759/cureus.34306

**Published:** 2023-01-28

**Authors:** Hani S Almugti, Shahd AlMarei, Razan M Jurebi, Meqren K Almutiri, Basil Alghamdi, Abdullah S Alghamdi, Hamad H Alhajry, Sarah M Al-Helali, Ashwaq H Alzaidi, Yahya S Alzahrani, Mamdouh H Al-Mutairy, Abdulrahman Jurebi, Ammar Alshareef, Abduljalil Almarzooq, Marwa Q Alsaedi

**Affiliations:** 1 Primary Health Care, Ministry of National Guard - Health Affairs, King Abdullah International Medical Research Center, King Saud bin Abdul-Aziz University for Health Sciences, Jeddah City, SAU; 2 Medicine, Jazan University, Jazan, SAU; 3 Pharmacy, King Khalid University Hospital, Riyadh, SAU; 4 Medicine, Al-Baha University, Al-Baha, SAU; 5 Medicine, Dar Al-Uloom University, Riyadh, SAU; 6 Medicine, Al-Rayan University, Medinah, SAU; 7 Pharmacy, United Pharamcies' Company, Taif, SAU; 8 Nursing, Central Second Health Cluster, Riyadh, SAU; 9 Medicine, Batterjee Medical College, Jeddah, SAU; 10 Internal Medicine, King Faisal Hospital, Makkah, SAU; 11 Medical Affairs, King Fahad Hospital, Hufof, SAU; 12 Primary Healthcare, Ministry of Health Holdings, Madinah, SAU

**Keywords:** dermatological diseases, kingdom of saudi arabia (ksa), surveillance system, compliance, primary healthcare worker

## Abstract

Background: Control and prevention of infectious diseases has been a primary health mandate. The reporting system is a vital step in preventing and controlling of these diseases. Most important, healthcare workers who have a responsibility to report must be aware of this responsibility. The present study aimed to improve the compliance of primary healthcare workers against reportable tropical and non-tropical dermatological diseases.

Objective of the study: The objective was to assess the knowledge, skills, and practice of primary healthcare workers in Saudi Arabia regarding the surveillance system of reportable tropical and non-tropical dermatological diseases using an assessment tool featuring closed-ended questions. As a secondary objective, this study assessed the satisfaction of primary healthcare workers with the surveillance system.

Subjects and methods: Through a cross-sectional design, the study used an electronic self-administered questionnaire targeting the primary healthcare workers who met the inclusion criteria through a non-probability sampling technique.

Results: By the end of the study period, data had been collected from 377 primary healthcare workers. Slightly more than half of them worked for the ministry of health facilities. In the last year, the vast majority (88%) of participants did not report any infectious diseases. Poor or low knowledge was reported by almost half of the participants concerning which dermatological diseases should be notified immediately on clinical suspicion or routinely on a weekly basis. Clinically and in response to the skills assessment, 57% of the participants had lower skills scores in detecting and identifying the skin ulcer of leishmania. Half of the participants were less satisfied with the feedback after their notification and considered the notification forms complicated and time-consuming, especially with the usual high workload in primary healthcare centers. Furthermore, the observed significant differences (p < 0.001) in knowledge and skill scores were demonstrated with female healthcare workers, older participants, employees from the Ministry of National Guard Health Affairs, and workers with more than ten years of experience.

Conclusion:The present study has shown the limitations of public health surveillance due to underreporting and lack of timeliness. The dissatisfaction of study participants with feedback after the notification step is another finding that demonstrates the need for collaboration among public health authorities and healthcare workers. Fortunately, health departments can implement measures to improve practitioners' awareness through continuous medical education and providing frequent feedback to overcome these hurdles.

## Introduction

In the context of public health, the term “surveillance” was first applied to the close observation of people who, due to exposure, were at risk of acquiring highly contagious infectious diseases that had been eradicated or controlled in a specific region or among a particular population [1]. This term has recently been broadened to include any outcome, hazard, or exposure in addition to disease [2]. The primary goal of public health surveillance is assuring the effectiveness of the implemented preventive measures and, if required, improving them [2].

The typical data sources for infectious diseases surveillance come from individuals, the environment, and healthcare facilities using a limited number of methods, such as environmental monitoring, surveys, notifications, and registries. Information regarding epidemiology is frequently obtained from notifications [1]. As shortly as an infectious disease is detected (or suspected), the local health authority should be notified, and then accordingly, it becomes their responsibility to implement suitable control measures [3].

Globally, over the past five years, several emerging and re-emerging infectious diseases were detected, investigated, and rapidly contained, including COVID-19, Monkeypox, Ebola virus disease (EVD), severe acute respiratory syndrome (SARS), Middle East respiratory syndrome (MERS), and Zika virus disease [4,5]. The occurrence of such diseases highlights the need for effective and advanced public health surveillance at national and international levels.

Saudi Arabia is a developing Islamic Middle Eastern country. Aside from many Muslim visitors coming for the Hajj and Umrah [6], there is a high percentage of the workforce from tropical lands or temperate countries harboring tropical notifiable diseases [7]. For that, and in response to these public health challenges, in 2012, Saudi Arabia worked to develop the Saudi Health Electronic Surveillance Network system (HESN), which was introduced to support the healthcare system's ability to tackle ongoing global health issues and promote national health security [8].

Several studies [8,9] indicated that the surveillance system's effectiveness is directly associated with the ability of healthcare workers to comprehend the importance of reporting cases. Furthermore, underreporting of cases by healthcare workers was addressed in previous studies as a contributing factor to the impairment of the surveillance system and health security [10,11]. Although most of the required reportable tropical and non-tropical dermatological illnesses can be reported based on clinical suspicion [12], numerous studies [11,13] have linked the lack of compliance to reporting with the resources and infrastructure of the healthcare system.

Particularly for infectious dermatological diseases, the availability of data to assess compliance with the Saudi surveillance system at the individual level (HCWs level) has been limited up to this point. Implementing a reliable and effective electronic health system requires sufficient and advanced infrastructure in addition to trained and competent healthcare workers who can identify the clinical spectrum of clinical cases and proceed with reporting based on clinical suspicion to the local health authority. In light of this, this study aims to improve the notification practice against reportable dermatological diseases among primary healthcare providers in Saudi Arabia by identifying the factors associated with their compliance.

## Materials and methods

Aim of the study

The study aimed to improve the compliance of primary healthcare workers against reportable tropical and non-tropical dermatological diseases. The primary objective was to assess the knowledge, skills, and practice of primary healthcare workers in Saudi Arabia regarding the surveillance system of reportable tropical and non-tropical dermatological diseases using an assessment tool featuring closed-ended questions. The secondary objective was to assess the satisfaction of primary healthcare workers with the surveillance system.

Study area/setting

The study was carried out in primary health centers in Saudi Arabia. Primary healthcare centers serve as the first line of patient contact with the healthcare system, and the frontline care for reporting notifiable diseases.

The current research participants were approached through an electronic questionnaire that was distributed on Facebook, WhatsApp, and Twitter. These online platforms are considered the most popular social media used by the Saudi nation.

Study design and study subjects

The subjects of the current cross-sectional study were the primary healthcare workers who were working at Saudi primary healthcare centers under the following health institutes (Ministry of Health, Ministry of National Guard, and health services of Ministry of Defence) and had the job title of physician, nurse, public health specialist, or lab worker. However, students and interns from the medical and health college were excluded from this study. 

Sample size and sampling technique

Using Epi Info statistical software, version 7.2, the sample size of 377 primary healthcare providers was calculated at a confidence level of 95% and a 5% margin of error. This sample size was estimated at 21,000 [14], the total number of primary healthcare providers in Saudi Arabia who met the inclusion and exclusion criteria.

From the Saudi high-capacity Healthcare institutes (Ministry of Health, Ministry of National Guard, and health services of Ministry of Defense), the primary healthcare workers were invited to fill up the questionnaire through non-probability sampling (snowballing sampling technique).

Data collection methods, instruments used, and measurements

Variables of the Study

Dependent variables: The primary healthcare workers' mean score of both the knowledge and skills regarding the surveillance system of reportable tropical and non-tropical dermatological diseases, in addition to the mean score of their satisfaction with the surveillance system in general.

Independent variables: Age, gender, nationality, profession, length of experience, type of primary health care center (Ministry of Health, Ministry of National Guard, and health services of Ministry of Defense), and history of obtaining training courses on Notifiable Communicable Diseases.

Compliance of Healthcare Workers

From the Oxford dictionary [15], compliance means procedures that must be followed to ensure complete adherence to the law. In the present study, compliance with the surveillance system is defined as the required knowledge and skills that primary healthcare workers should have and the clinical practices, they should do to follow the requirement of reporting and notification system of diseases.

Definition of Tropical Notifiable Dermatological Diseases

Skin diseases that most frequently diagnosed among immigrants and travelers in non-tropical countries. Acquiring these diseases occur in tropical and subtropical locations [7]. 

Operation Definition of Dermatological Diseases (Tropical and Not Tropical) in This Study

Table 1 illustrates the infectious diseases that have skin manifestations in their standard clinical definitions corresponding to the Saudi public health surveillance system [12], such as measles, chickenpox, Monkeypox, dengue fever, scabies, and leishmaniasis.

**Table 1:** Notifiable skin diseases in the Saudi public health surveillance system

Class I Disease ( Diseases that must be notified within 24 Hours, Just with Suspicion)	Class II Diseases ( Diseases that should be notified weekly)
Plague Cholera Yellow fever Typhus Anthrax Diphtheria. Acute flaccid paralysis AFP as Polio, other suspected polio cases like Gillian Barrie syndrome & transverse myelitis Neonatal tetanus Measles Rubella Tuberculosis Human immunodeficiency virus (HIV) Meningococcal meningitis Viral hemorrhagic fevers (Crimean- Congo fever, rift valley fever, dengue fever, Ebola, Lasa fever & west Nile fever). Food poisoning Middle East respiratory syndrome coronavirus (MERS-COV) Schistosomiasis.	Hepatitis A. Hepatitis B. Hepatitis C Chickenpox Mumps. Typhoid &paratyphoid. Salmonellosis. Whooping cough. Congenital rubella. unspecified hepatitis Rabies. shigellosis hydatid disease, hemolytic uremic syndrome gonorrhea Scabies. Tetanus other types. Brucellosis Amoebic dysentery. Puerperal fever. Syphilis. Malaria Leprosy

Questionnaire

The questionnaire of this study has been obtained with permission from previous research [11]. After reviewing the contents and items of the questionnaire, some modifications were made to make it suitable for the current reporting system in Saudi Arabia and to meet the objectives of the present study. In order to ensure the content and face validation, this questionnaire was reviewed by health professionals from dermatology, infection control, and public health who were invited by email. In addition, a pilot study with 14 primary healthcare workers was carried out to ensure the questionnaire's reliability, and clarity, and to measure the time required to fill out the questionnaire. Cronbach's alpha coefficients ranged from 0.76 to 0.8, indicating good reliability.

However, the questionnaire of this study is an online self-administered and consists of five sections, the first section included the socio-demographics and occupational data, the second section included the practice assessment related to the Notifiable Diseases Surveillance System, the third section was about the knowledge assessment regarding the Notifiable Diseases Surveillance System, the fourth section was about the assessment of the required skills for the Notifiable Diseases Surveillance System, and the last section Included the assessment of primary healthcare workers' satisfaction with the Notifiable Diseases Surveillance System.

Data management and analysis plan 

The Windows-based SPSS statistical software suite was used for data entry and analysis (version 20.0; IBM Corp., Armonk, NY, USA). Data entry and coding stages were performed to enhance the data quality. Data were presented using frequencies and percentages for qualitative variables, whereas means and standard deviations were used for quantitative variables. The Chi-square test was used between participants' answers and other variables to record the statistically significant.

## Results

Demographic and occupational information

Of the invited primary healthcare workers, 377 answered the online questionnaire, (Table 2) shows that slightly more than half of them worked for the ministry of health. The great majority of participants (94%) were less than 40 years old. Almost two-third of the respondents were physicians, non-Saudi, and had work experience of more than five years.

**Table 2:** Demographic and Occupational characteristics of participants (n=377)

Demographic characteristics	Frequency (n)	Percent (%)
Age category		
20 - 30 years	94	25
31 - 40 years	260	69
41 - 50 years	23	6
Gender		
Male	212	56
Female	165	44
Nationality		
Saudi	154	41
Non- Saudi	223	59
Health Sectors		
Ministry Of Health	213	56
Ministry of National Guard Health Affairs	118	31
Health services of the Ministry of Defense	46	12
Job title		
Physician	283	75
Nurse	94	25
Length of Experience		
Less than five years	117	31
From five to ten years	141	37
More than ten years	119	32

Clinical practices related to the notifiable diseases surveillance system

**Figure 1:**
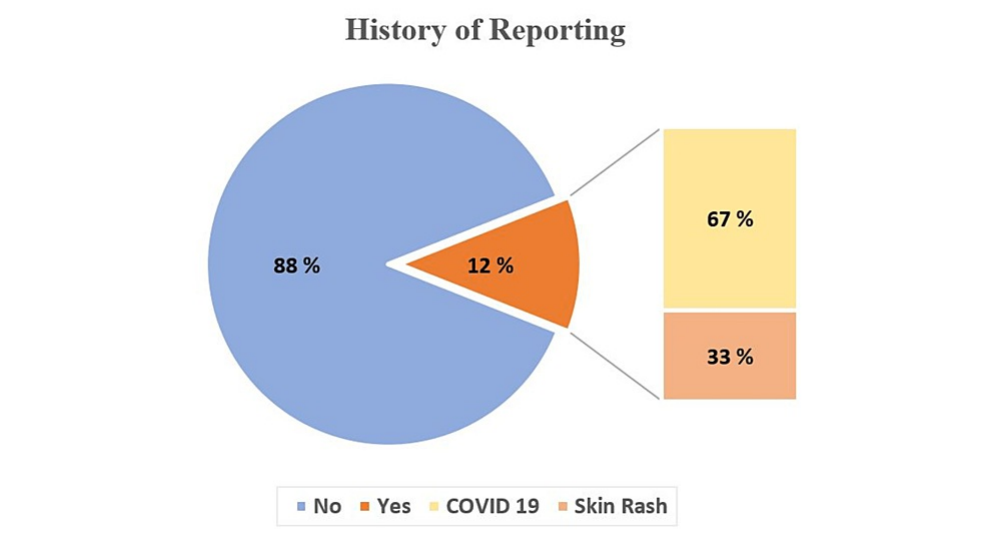
The participants' reporting history for the notifiable diseases (n=377)

**Figure 2:**
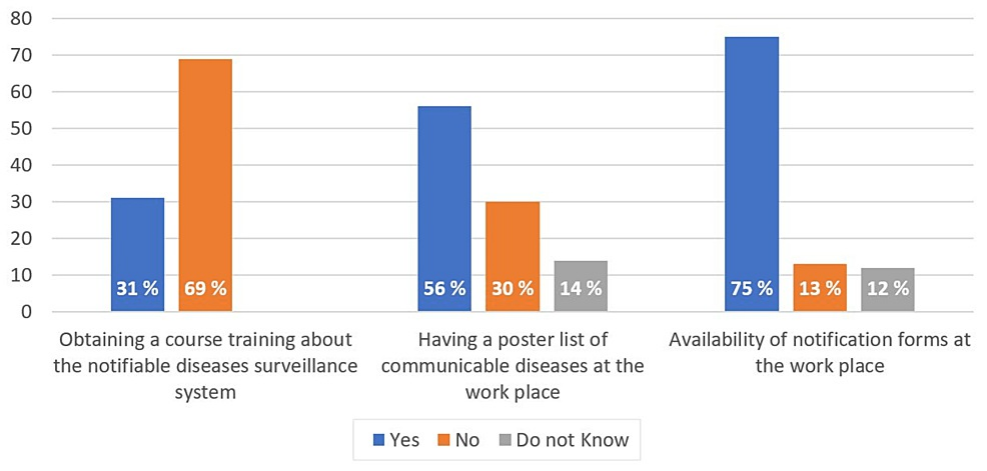
The supportive structure of the notifiable diseases surveillance system (n=377)

As shown in Figure 1, a small percentage of participants (12%) reported notifiable diseases in the last year; two-thirds of the reported infectious diseases were COVID-19. On the other hand, Figure 2 illustrates that approximately one-third of the participants had a training course about the national surveillance systems of communicable diseases. From the participants' daily practice, two-thirds of participants stated that the notification forms were available at their facility. Furthermore, a poster about the list of reportable communicable diseases was available at the workplace of slightly more than half of the participants.

 

The assessment of knowledge and skills regarding the notifiable diseases surveillance system

In response to the knowledge assessment questions (Table 3), most of those surveyed (66%) indicated that they understood the need for a surveillance system. However, slightly half of the participants reported poor or low knowledge concerning which dermatological diseases should be notified immediately on clinical suspicion or routinely on a weekly basis.

On the other hand, the participants' skills assessment in Table 3 demonstrates poor clinical skills in diagnosing skin ulcers of cutaneous Leishmania among slightly more than half of the participants (57%). However, almost half of the participants reported that they had a good level of clinical skills to diagnose skin rashes (44%), had a good clinical ability to differentiate between scabies, skin dermatitis, or folliculitis (45%), and had good skills to diagnose dengue fever (50%).

**Table 3:** The assessment of knowledge and skills regarding the notifiable diseases surveillance system (n=377)

Item		No (%)					Score (max=5)	
		Poor or Low		Neutral	Good or High		Mean ± SD	Median
Knowledge assessment								
Understand the need for a notifiable disease surveillance system.		72 (19%)		57 (15%)	248 (66%)		3.8 ± 0.4	4
Know the dermatological infectious diseases that should be notified immediately on clinical suspicion.		181 (48%)		100 (27%)	96 (25%)		2.8 ± 0.7	3
Know which dermatological infectious diseases should be notified within 24 hours of diagnosis.		119 (32%)		117 (31%)	141 (37%)		3 ± 0.4	3
Know which dermatological infectious diseases can be notified weekly.		189 (50%)		71 (19%)	117 (31%)		3.1 ± 1.12	3
Skills assessment								
Ability to clinically differentiate between the skin rashes of measles, chickenpox, and Monkeypox	72 (19%)		141 (37%)			164 (44%)	3.24 ± 0.7	3
Ability to clinically differentiate between scabies, skin dermatitis, or folliculitis.	76 (20%)		131 (35%)			170 (45%)	3.19 ± 0.7	3
Ability to clinically diagnose dengue fever	96 (26%)		93 (24%)			188 (50%)	3.24 ± 0.8	3
Ability to clinically diagnose skin ulcer of cutaneous Leishmania	215 (57%)		110 (29%)			52 (14%)	2.9 ± 0.8	3
Ability to access the latest protocols and guidelines on notifiable diseases	38 (10%)		98 (25.5%)			241 (64%)	3 ± 0.8	3
Ask for Consultation if there is uncertainty about diagnosing or managing any notifiable disease	109 (29%)		118 (31%)			150 (40%)	2.9 ± 0.8	3

Participants' satisfaction with the notifiable diseases surveillance system

Table 4 illustrates that almost half of the participants (44%) found that the forms used to notify diseases were time consumed and not simple to be understood. Moreover, 46% agreed that the high workload was an obstacle to notifying the diseases during clinical practice. Concerning participants' satisfaction with action taken after notification, 64% of participants were uncertain if the department provided feedback after their notification, and almost half of them (54%) were unsure if the outbreak response was on time.

**Table 4:** Participants' satisfaction with the notifiable diseases surveillance system (n=377)

Participants' satisfaction with the surveillance system	No (%)			Score (max=5)	
	Agree/Strongly agree	Uncertain	Disagree/Strongly disagree	Mean ± SD	Median
The form used to notify diseases is easy to understand	24 (6%)	188 (50%)	165 (44%)	2.6 ± 0.6	3
The form used to notify diseases takes a long time to fill in	165 (44%)	117 (31%)	95 (25%)	2.8 ± 0.8	3
A high workload prevents me from notifying diseases	175 (46%)	119 (32%)	83 (22%)	2.9 ± 0.8	3
The department provided no feedback on notifiable diseases.	19 (5%)	241 (64%)	117 (31%)	± 0.79	3
Outbreak response teams do respond timely to most outbreaks	110 (29%)	203 (54%)	64 (17%)	2.7 ± 0.7	3

Relation between the knowledge and skill score of participants and their demographic and occupational characteristics

Although there was no statistical significance of association between the variables of nationality and job title with the knowledge score, Table 5 demonstrates an association with statistical significance between participants' knowledge score and other demographic and occupational characteristics (p < 0.05). Higher knowledge scores were demonstrated among female healthcare workers, older participants, employees from the Ministry of National Guard Health Affairs, workers with work experience of more than ten years, and participants who had a course on the notifiable diseases surveillance system.

**Table 5:** Relation between the knowledge score of participants and their demographic and occupational characteristics (n=377) (*) Statistically significant at p<0.05

	Knowledge score (max=20)		
Demographic and Occupational characteristics	Median	Median test Statistic	p-value
Age category			
20 - 30 years	5		
31 - 40 years	10		
41 - 50 years	7	6.9	0.001*
Gender			
Male	7		
Female	10	0.57	0.001*
Nationality			
Saudi	8		
Non- Saudi	7	4.6	0.063
Health Sectors			
Ministry of Health	8		
Ministry of National Guard Health Affairs	12		
Health services of the Ministry of Defense	7	0.2	0.001*
Profession			
Physician	8		
Nurse	7.5	5.057	0.08
Length of Experience			
Less than five years	6		
From five to ten years	8		
More than ten years	10	14.7	0.003*
Having a training course bout the notifiable diseases surveillance system			
Yes	12		
No	7	10.08	0.002*

The results, as shown in Table 6, indicate that participants with more than ten years of experience, female, and employees from the Ministry of National Guard Health Affairs had higher median skill scores with statistical significance (p 0.05) in relation to their demographic and occupational characteristics. Interestingly, there were no differences between those who had a training course about the notifiable diseases surveillance system and who had not.

**Table 6:** Relation between the skill score of participants and their demographic and occupational characteristics (n=377) (*) Statistically significant at p<0.05

	Skill score (max=30)		
Demographic and Occupational characteristics	Median	Median test Statistic	p-value
Age category			
20 - 30 years	16		
31 - 40 years	19		
41 - 50 years	24	21	0.0001*
Gender			
Male	18		
Female	20	0.182	0.01*
Nationality			
Saudi	18		
Non- Saudi	19	8.9	0.2
Health Sectors			
Ministry of Health	18		
Ministry of National Guard Health Affairs	21		
Health services of the Ministry of Defense	19	24	0.001*
Profession			
Physician	18		
Nurse	19	23.6	0.4
Length of Experience			
Less than five years	18		
From five to ten years	18		
More than ten years	23	3.05	0.001*
Having a training course bout the notifiable diseases surveillance system			
Yes	18		
No	19	19	0.3

## Discussion

A surveillance system in the primary healthcare setting is essential to an effective public infection prevention and control program. In the current study, we assessed the knowledge, skills, and practice of healthcare workers in Saudi primary healthcare centers regarding the surveillance system. The majority of study participants (56%) in this study were from the Ministry of Health, which is consistent with the fact that the Saudi Ministry of Health and its network of primary care facilities provide the majority of the country's public health services [16].

Infectious disease underreporting is a major challenge for any surveillance system. Previous research [17] attributed that to various reasons, including undetectable asymptomatic diseases, substandard public health infrastructures, and government censorship. Similar to other studies' findings [11,18], the vast majority (88%) of participants in the current study did not report any infectious diseases, though they worked at primary healthcare facilities where it was expected to encounter a variety of infectious cases that required reporting. Furthermore, about two-thirds of participants have yet to attend a surveillance training course regarding reporting and other surveillance system processes, which is considered another finding that might explain the non-compliance with communicable disease reporting. Workplace education was presented in a previous study [19] as a practical solution to improve the reporting rate.

Early outbreak detection and response are characteristics of a highly effective disease control and prevention program. Given this, timeliness is recognized as a key indicator in assessing the effectiveness of infectious disease surveillance systems [20,21]. The results of the present study indicate a knowledge gap among the participants, as almost half reported insufficient knowledge of which dermatological diseases should be reported immediately or weekly. However, this finding further supports the need for workplace education for the best professional practices and surveillance improvement.

For each targeted infectious disease, establishing a case definition is imperative [22] in order to standardize the public health surveillance system. Although most national and international organizations provide lists of uniform case definitions for diseases that require to be reported, some diseases, including cutaneous leishmaniasis [23], are challenging to identify clinically due to their varied and broad clinical presentation. This might explain the present study's finding of poor clinical skills among slightly more than half of the participants in diagnosing skin ulcers of cutaneous Leishmania. A prior study, however, recommended linking the clinical presentations of diseases with other related epidemiological data on ongoing outbreaks in order to improve the detection of these diseases [24].

The second objective of the present study was to assess participants' satisfaction with the surveillance system. Almost half of the participants considered the notification forms complicated and time-consuming. Moreover, as per the current study participants, the high workload was the justification behind the low compliance with reporting. However, these findings have frequently been reported in previous studies [17,18] and demonstrate the need for leader support to address these ongoing issues from the related authority.

After the notification step, distributing or disseminating the relevant findings from the health authority is essential in public health surveillance. It is considered a motivational factor that demonstrates the rapid response and reinforces the importance of participating in a meaningful public health activity. The results of this study indicate that at least half of the participants were less satisfied with the feedback after their notification and were unsure if the outbreak response was on time. This finding supports the need for maintaining a collaboration among the public health and medical communities, which improves the reporting of diseases to health authorities.

According to the results of the present study, the observed significant differences in knowledge and skill scores were demonstrated with female healthcare workers, older participants, employees from the Ministry of National Guard Health Affairs, and workers with work experience of more than ten years. The relatively high scores of knowledges and skill are explainable for the participants of older age as they have more experience in reporting and notification practices of the cases. Furthermore, the recorded high score by the female participants is somewhat surprising since this difference has yet to be found elsewhere. On the other hand, the relatively high score among employees from the Ministry of National Guard Health Affairs is difficult to explain, but it might be related to the structure of the national guard health system, which provides health care to a specific target of the population through a lower number of primary healthcare centers compared with other health sectors, thus will facilitate monitoring, evaluating and improvement of the surveillance system.

This study has several limitations. First, it targeted the primary healthcare level, which is a part of several levels in the Saudi health system; however, secondary, tertiary, and private health sectors were not included. This had an impact on the generalizability of this study. Second, although gathering information from the participants themselves about their perception, satisfaction and practice always come with positive input in evaluation studies, the self-administered online questionnaire may overestimate the knowledge assessment because of the easy access to outside resources. Third, this study's cross-sectional nature provides a picture of practices at the time of the study; this may lead to an underestimating of the number of reported cases in previous years or other related practices. Lastly, the questions of the present study were for reportable dermatological diseases because have the ability to be detected clinically and reported on suspicion without waiting for the lab results; however, we could not comprehensively focus on these diseases as the surveillance system including a variety of diseases with the broad clinical presentation and all of them have national and international importance and required to be reported.

## Conclusions

The present study has provided valuable insights into the limitations of public health surveillance due to underreporting and lack of timeliness, which compromise its usefulness. The present study's findings show that a considerable percentage of participants did not undergo training courses regarding the required knowledge and practice of public health surveillance. Moreover, the dissatisfaction of study participants with feedback after the notification step is another finding that demonstrates the need for collaboration among public health authorities and healthcare workers. However, periodic evaluation of public health Surveillance is required to ensure that it serves a valuable public health function and meets its objectives. Surveillance might have limitations; fortunately, health departments can implement measures to improve practitioners' awareness through continuous medical education and providing frequent feedback to overcome these hurdles.

## Appendices

The questionnaire of this study is an online self-administered and consists of five sections as described in the following table:

**Table 7:** The contents of the five sections of study's questionnaire

Questionnaire		
includes Age, Gender, Nationality, profession, length of experience, type of primary health care center (Ministry of Health, Ministry of National Guard, or health services of Ministry of Defense), and obtaining training courses on Notifiable Communicable Diseases	Socio-demographics data	First section
Includes the assessment of current practice corresponding to the notification and reporting requirement. This section consists of the following four questions: 1) Did you ever receive training on Notifiable Communicable Diseases? 2) Do you have a list of communicable Diseases that should be notified in your facility/practice? 3) Do you have notification forms available in your facility/practice? 4) Did you notify any notifiable infectious diseases in the last year? If yes, which notifiable infectious diseases did you report?	Practice assessment related to the Notifiable Diseases Surveillance System	Second section
Includes the assessment of primary healthcare workers' level of knowledge regarding the Notifiable Diseases Surveillance System. This section consists of a list of four (4) facts of information. For each, the participant will indicate the current level of knowledge to perform the task on a scale of 1-5 ( with 1 being Low knowledge, needing more support or training) and five being (Very known, no support or training needed): 1) I know and understand the need for a notifiable diseases surveillance system 2) I know which dermatological infectious diseases should be notified immediately on clinical suspicion 3) I know which dermatological infectious diseases should be notified within 24 hours of laboratory confirmation of diagnosis 4) I know which dermatological infectious diseases can be notified of on a weekly basis.	Knowledge assessment regarding the Notifiable Diseases Surveillance System	Third section
includes the assessment of primary healthcare workers' skills level regarding the Notifiable Diseases Surveillance System. This section consists of a list of six (6) skills. For each, the participant will indicate the current level of skill to perform the task on a scale of 1-5 ( with 1 being Low skill, needing more support or training) and five beings (Very high skill, no support or training needed): 1) I am confident and can clinically differentiate between the skin rashes of measles, chickenpox, and Monkeypox. 2) I am confident and can clinically differentiate between scabies, skin dermatitis, or folliculitis. 3) I am confident and can clinically diagnose dengue fever. 4) I am confident and can clinically diagnose skin ulcers of cutaneous Leishmania. 5) I can access the latest protocols and guidelines on notifiable diseases. 6) I know whom to consult if I am uncertain about diagnosing or managing any notifiable disease.	Required skills regarding the Notifiable Diseases Surveillance System)	Fourth section
Includes the assessment of primary healthcare workers' satisfaction with the Notifiable Diseases Surveillance System. This section consists of Five (5) statements on attributes of Notifiable Disease Surveillance. Participants will use the provided scale to state how strongly they agree or disagree with the statement. 1) The form used to notify diseases is easy to understand? 2) The form used to notify diseases takes a long time to fill in? 3) A high workload prevents me from notifying diseases 4) The department provided no feedback on notifiable diseases. 5) Outbreak response teams do respond timely to most outbreaks	Satisfaction with the Notifiable Diseases Surveillance System	Fifth section
